# Supplementary material for: Crystal structure of tripartite-type ABC transporter MacB from Acinetobacter baumannii
Source: Nat Commun. 2017 Nov 6;8:1336. doi: 10.1038/s41467-017-01399-2 (PMC5673888; doi:10.1038/s41467-017-01399-2)
Supplement: Supplementary file 1 — Supplementary Information [file 41467_2017_1399_MOESM1_ESM.pdf]

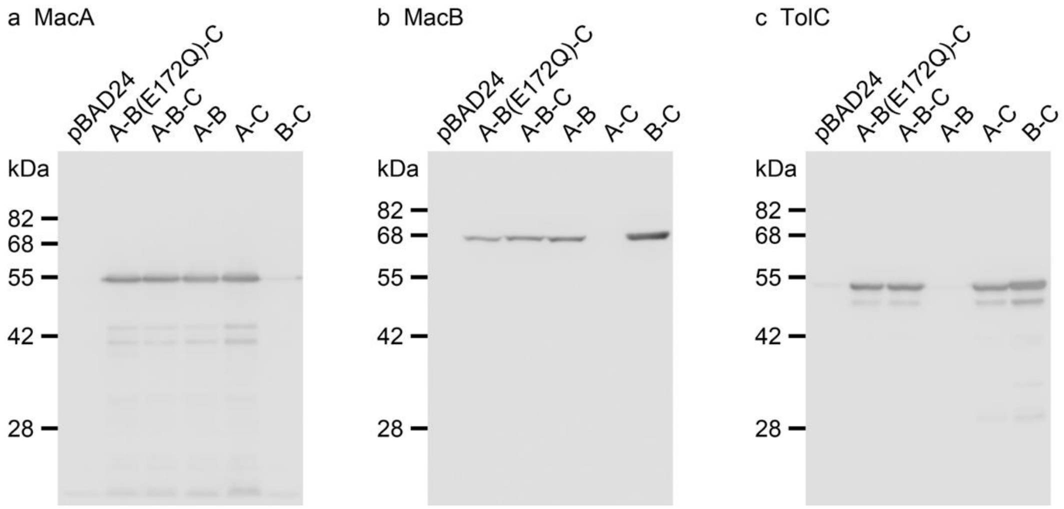

Supplementary Figure 1. Western blot showing expression of (a) MacA, (b) MacB and (c) TolC in *E. coli* W3104 Δ*acrAB* Δ*macAB* cells used in MIC measurements (Fig. 1, Table 1). Aliquots of the plasma membrane fractions (40 μg of total protein) were subjected to SDS-polyacrylamide electrophoresis. Proteins were detected on immuno blots using rabbit anti-MacA, rabbit anti-TolC, or mouse anti-MacB as the primary antibody, and horseradish peroxidase-labelled anti-rabbit or anti-mouse goat IgG as the secondary antibody, respectively. The migration of molecular mass markers (kDa) is indicated. Uncropped images are shown in Supplementary Figure 14.

a

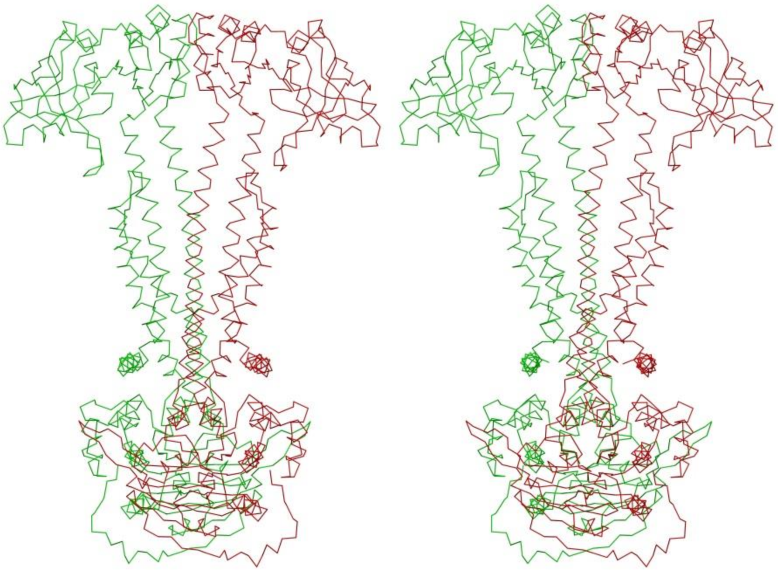

b

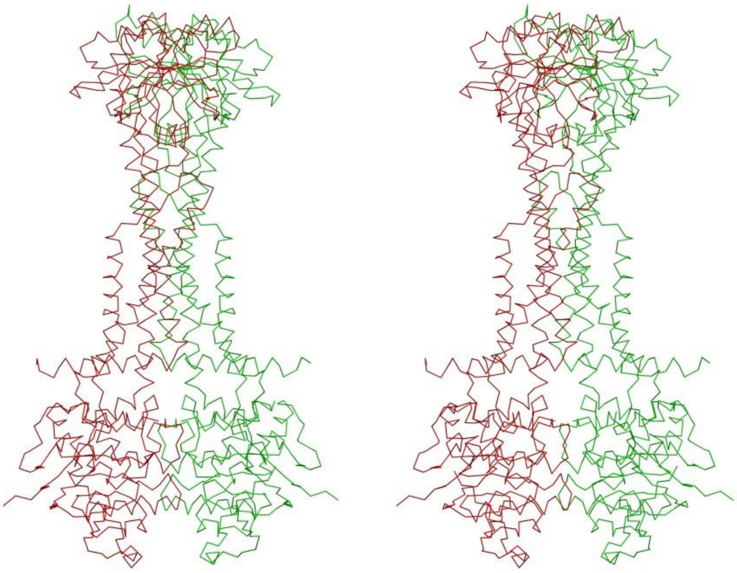

Supplementary Figure 2. Stereo view of the Cα traces of MacB, oriented as displayed in Figure 2a,b. The two protomers are also individually coloured (red and green) as in Figure 2a,b.

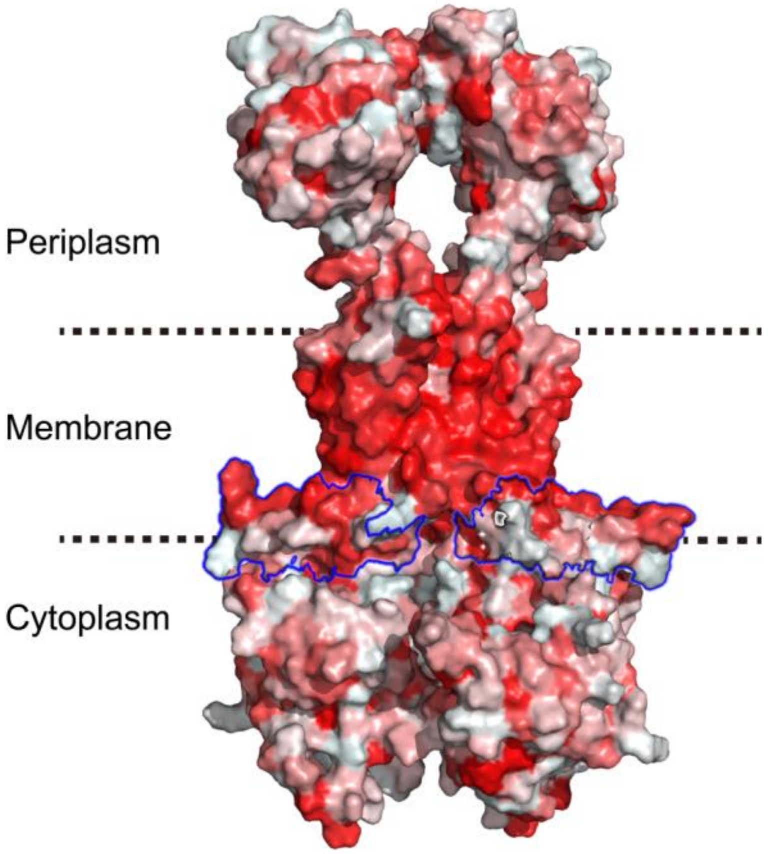

Supplementary Figure 3. Hydrophobic surface of the MacB dimer, viewed from the membrane plane. MacB dimer is shown in a surface representation (rendered in PyMOL) with hydrophobicity scale in white to red (high values are in dark red) according to Eisenberg[1]. Dotted black lines depict the approximate membrane boundaries. Helices rimmed with blue lines are elbow helices, which are partially embedded in the membrane.

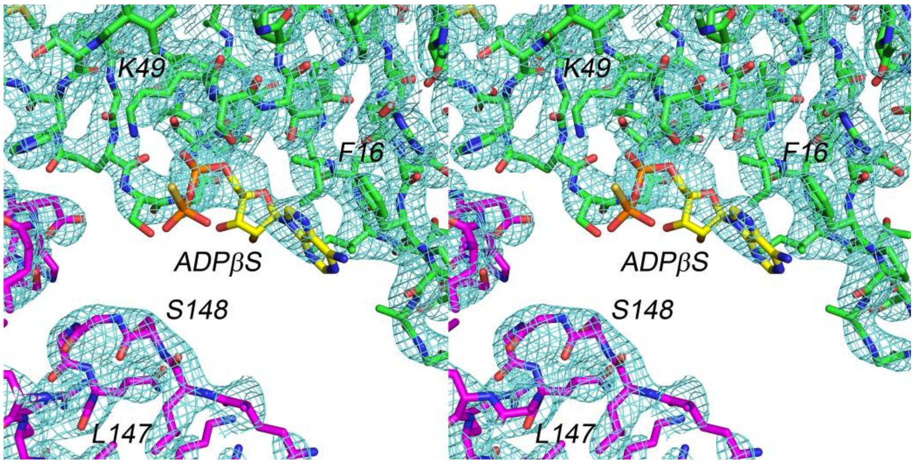

Supplementary Figure 4. Structure of the nucleotide-binding site in MacB. Stereo view of the nucleotide-binding site showing the fit of the structure model to the electron density map (2m*F*o-D*F*c at 1.5 σ). Carbon atoms in A monomer and B monomer of MacB dimer and ADPβS are coloured in green, magenta and yellow, respectively. Some of the functionally important residues are labelled individually.

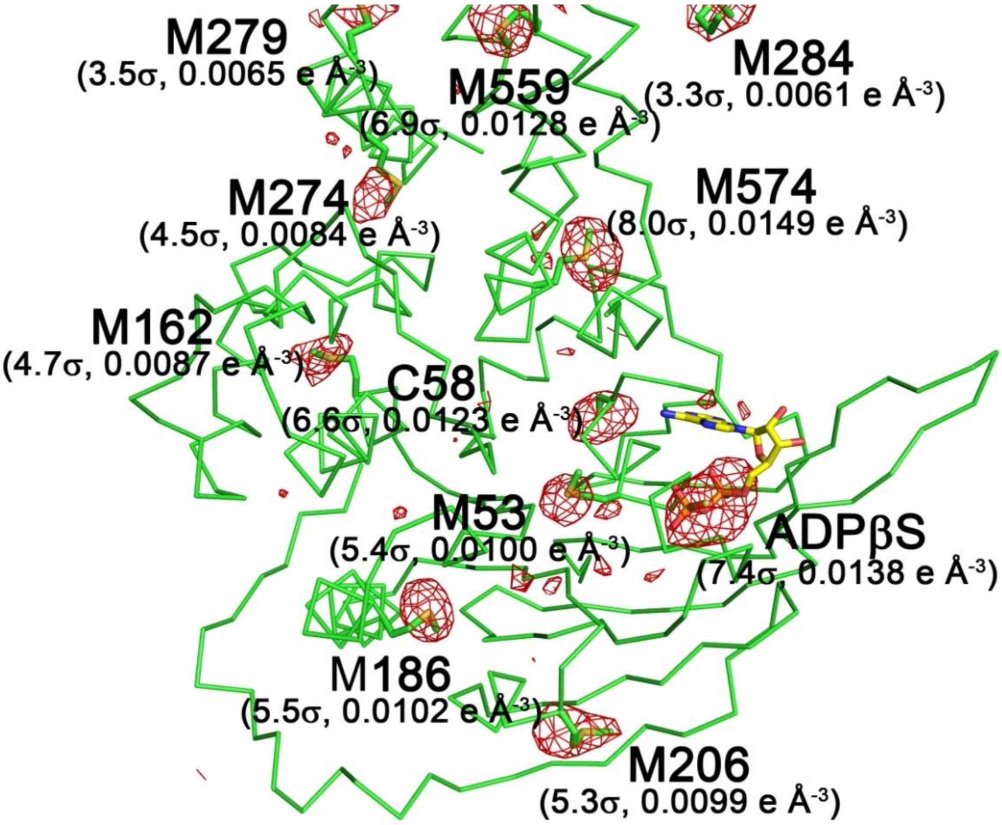

Supplementary Figure 5. Anomalous dispersion from sulphur and phosphor atoms in NBD. Main-chain tracing of monomer A of MacB is shown in ribbon representation (green). Side chains of methionine and cysteine residues and bound ADPβS molecule are shown in stick representation. The Anomalous Difference Fourier map contoured at 2σ (0.04736 e Å $^{-3}$) level for sulphur and phosphor atoms are depicted in red cages. Distinct anomalous dispersion from all of methionine and cysteine residues around NBD (M53, C58, M162, M186, M206) except N-terminal methionine is clearly observed. Sulphur and phosphor atoms of ADPβS are also clearly detected.

a

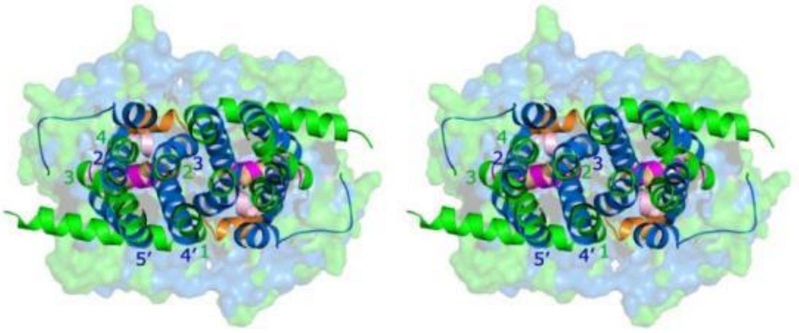

b

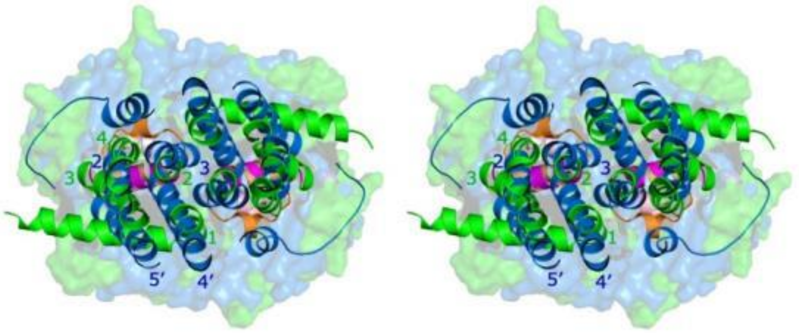

c

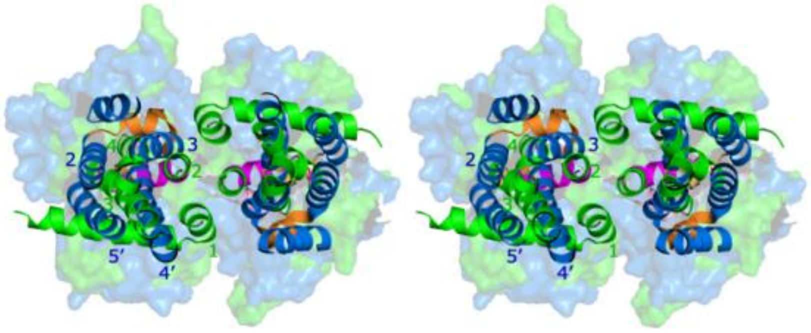

Supplementary Figure 6. Coupling helices have similar positions in MacB dimer compared to type-I exporters. NBDs are superimposed and shown in stereo-view. Cut view of the proximal half of TMD from distal side of the cell. (**a**) Sav1866 in outward-facing conformation (PDB accession code: 2ONJ) [2], (**b**) McjD in outward-occluded conformation (PDB accession code: 4PL0)[3], and (**c**) TM287-TM288 in inward-facing conformation (PDB accession code: 3QF4)[4] with the same colour as Figure 5a. TMs of each protein are numbered.TM 2, 3 of MacB correspond to TM4', 5' from the neighbouring monomer of Sav1866, respectively. TM4 of MacB corresponds to TM 2 of typical type-I ABC exporters. Locations of CHs are similar on each of the NBDs with important differences in connecting TMs due to unequal topologies

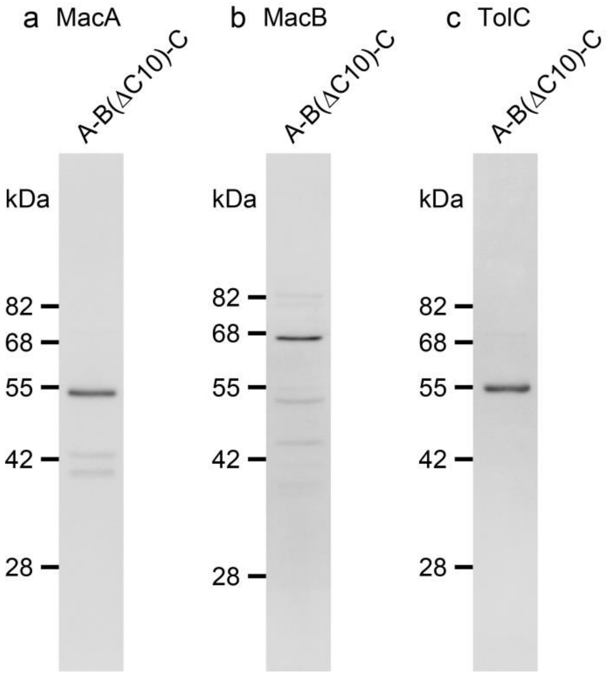

Supplementary Figure 7. Immuno blot showing expression of MacA, MacB (ΔC10) and TolC in *E. coli* W3104 Δ*acrAB* Δ*macAB* cells used in MIC measurements. Aliquots of plasma membrane fractions (40 μg of total protein) were subjected to SDS-polyacrylamide electrophoresis. Proteins were detected by immuno blotting using (**a**) rabbit anti-MacA, (**b**) rabbit anti-MacB, or (**c**) rabbit anti-TolC as the primary antibody, and horseradish peroxidase-labelled anti-rabbit IgG as the secondary antibody. Uncropped images are shown in Supplementary Figure 14.

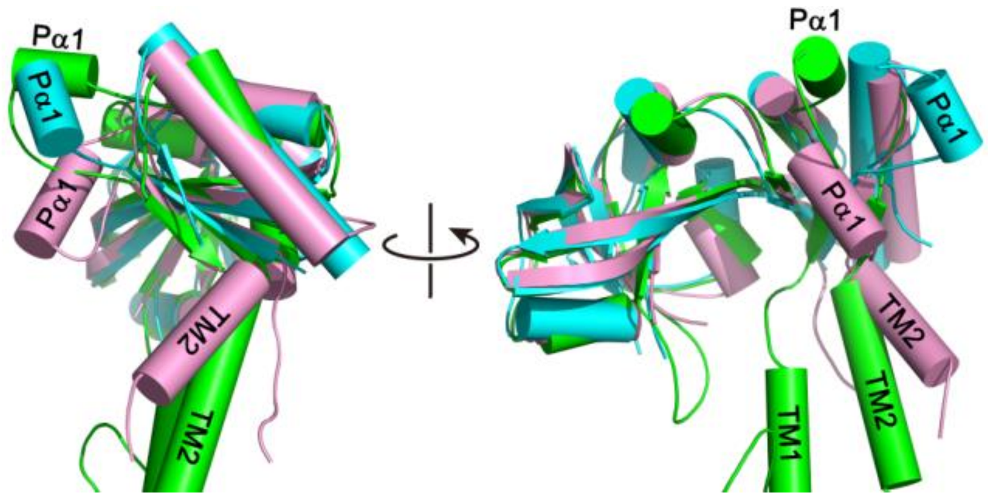

Supplementary Figure 8. Structure of MacB PLD. Structure comparison between full length MacB (green) and previously crystallized PLD from *Actinobacillus actinomycetemcomitans* MacB (pink, PDB accession code: 3FTJ)[5] and PLD of YknZ, a homologue from *Bacillus amyloliquefaciens* (cyan, PDB accession code: 5F9Q)[6] in two orientations, rotated by 90° around a vertical axis to the membrane plane. This comparison shows that the positions of Pα1 and connecting loop regions are extremely divergent.

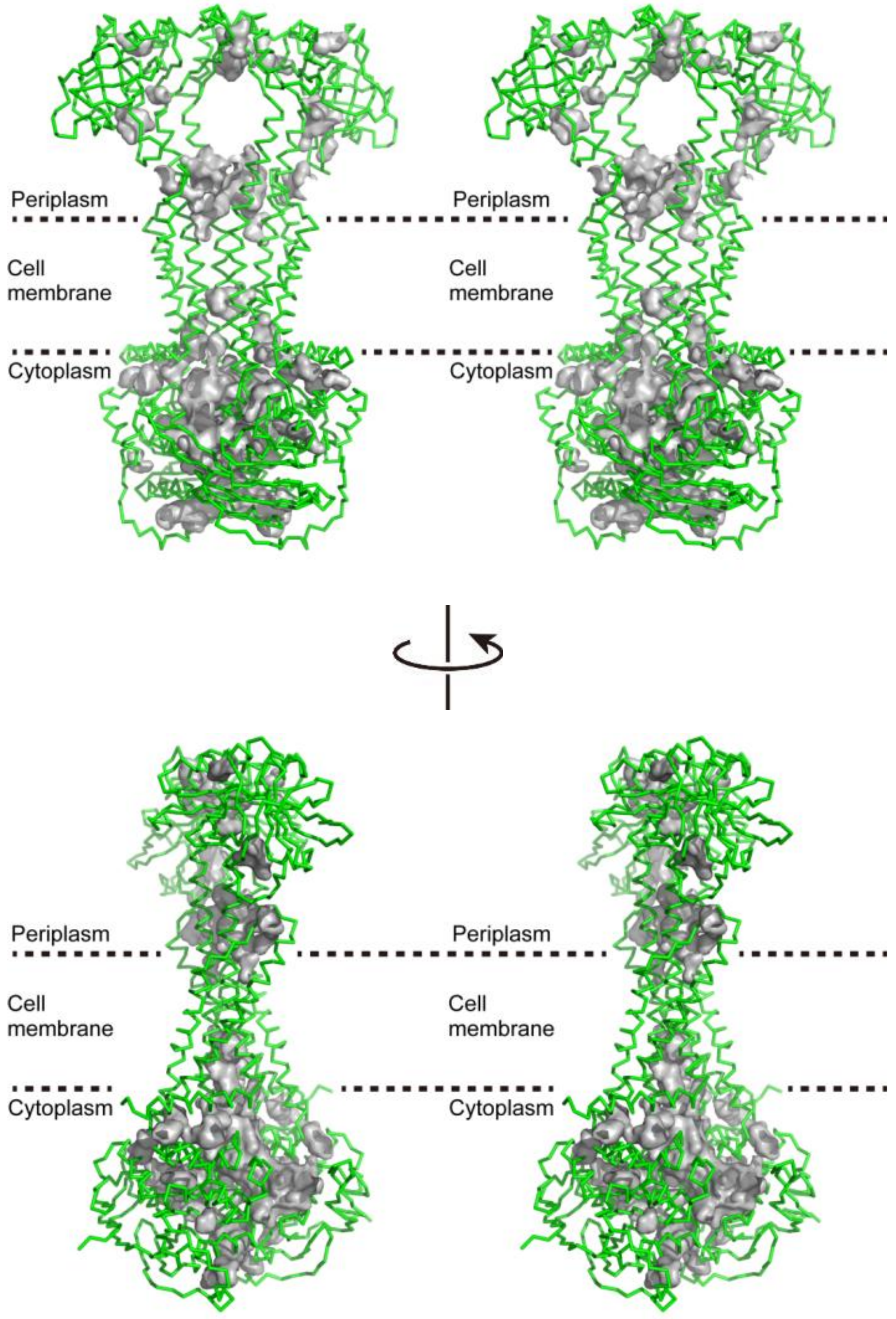

Supplementary Figure 9. Transmembrane cavity in MacB dimer, shown in stereo-view. Backbone traces of MacB dimer (green) in two orientations, rotated by 90° around a vertical axis to the membrane plane. Transmembrane cavity (in grey) was calculated with PyMOL with the cavity cull value of 29 Å.

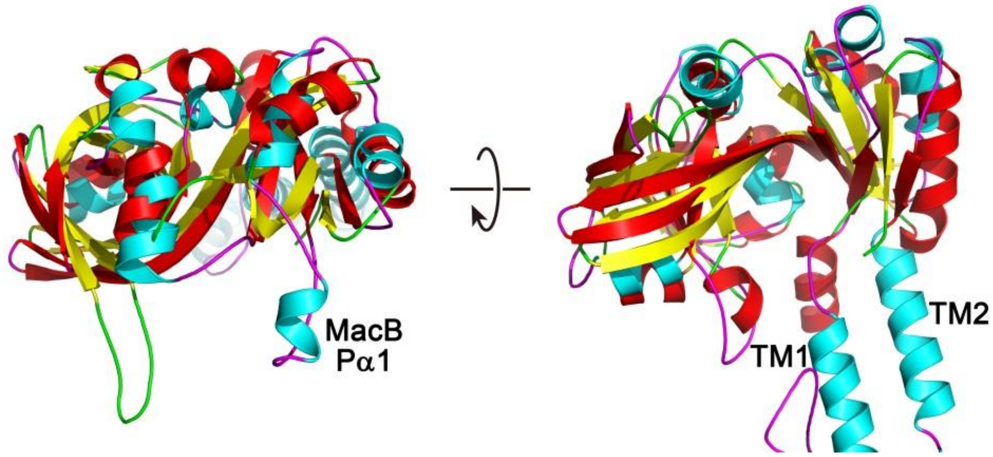

Supplementary Figure 10. Superimposition of PLDs of MacB and LolE (PDB accession code: 5UDF)[7]. (left) Top view and (right) side view are rotated by 90° around a parallel axis to the membrane plane. MacB is coloured in cyan for α-helices, red for β-sheets and magenta for loop regions, respectively. LolE is coloured in red for α-helices, yellow for β-sheets and green for loop regions, respectively. Protein folds in these structures are quite similar with high Z-score (Z=15.7) in program DALI[8,9].

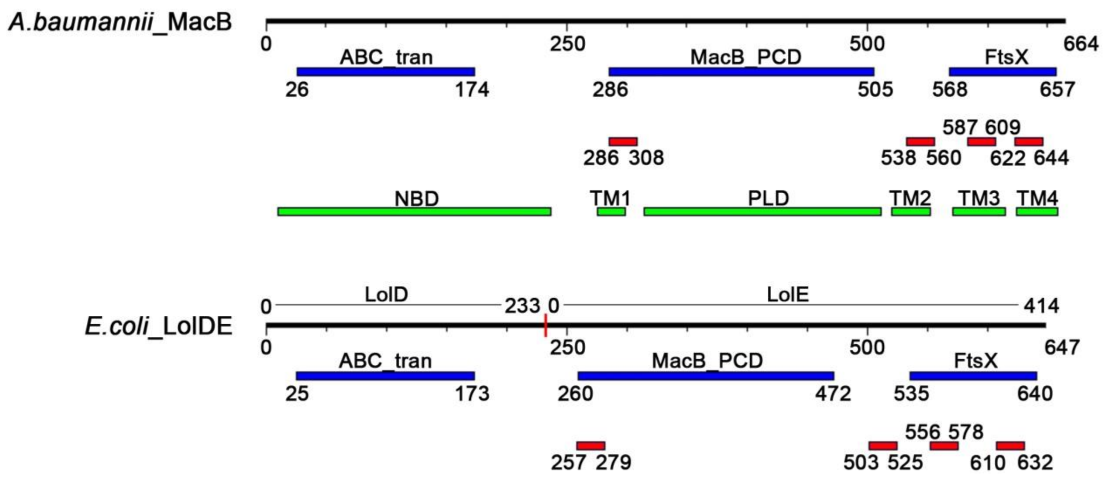

Supplementary Figure 11. Similarity of MacB to the lipoprotein ABC transporter LolCD$_2$E. LolD (233 amino acid residues) and LolE (414 amino acid residues) are expressed from overlapping genes to make the LolDE half-transporter of the full complex. Blue bars show the protein motifs annotated on Pfam, the protein family database[10]. Above the blue bars, domains and repeat families are indicated as ABC_tran; (PF00005), ATP-binding domain of ABC transporters, MacB_PCD; (PF12704), MacB-like periplasmic core domain, FtsX; (PF02687), FtsX-like permease family, respectively. Red bars show putative transmembrane helices predicted on the SOSUI server, the hydrophobicity- and amphiphilicity-based transmembrane helix prediction[11]. Green bars depict the structural elements in the MacB crystal structure which are summarised in Figure 3a. Thus, the two ABC half-transporters MacB and LolDE share similar structural elements.

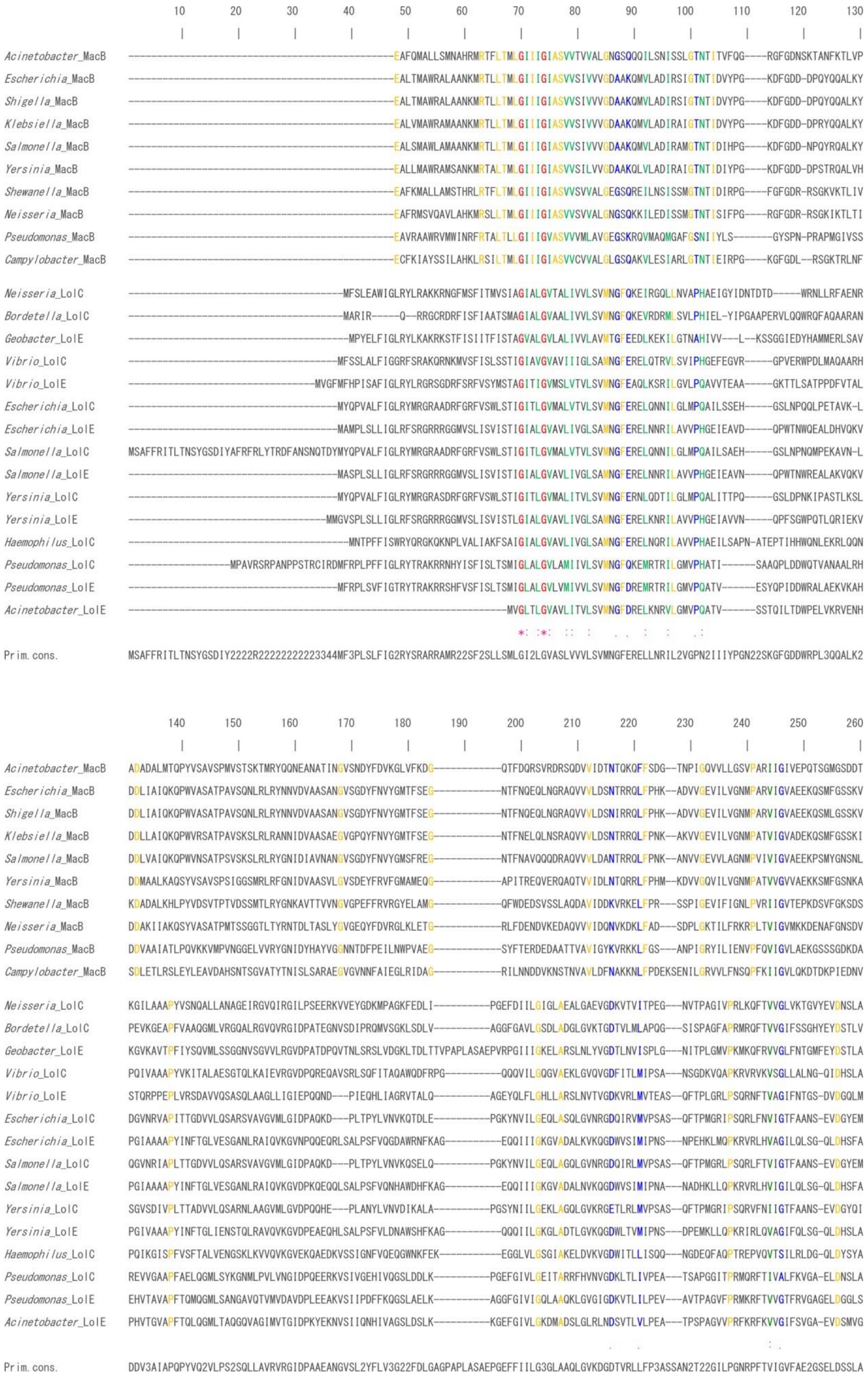
Acinetobacter_MacB
Escherichia_MacB
Shigella_MacB
Klebsiella_MacB
Salmonella_MacB
Yersinia_MacB
Shewanella_MacB
Neisseria_MacB
Pseudomonas_MacB
Campylobacter_MacB
Neisseria_LolC
Bordetella_LolC
Geobacter_LolE
Vibrio_LolC
Vibrio_LolE
Escherichia_LolC
Escherichia_LolE
Salmonella_LolC
Salmonella_LolE
Yersinia_LolC
Yersinia_LolE
Haemophilus_LolC
Pseudomonas_LolC
Pseudomonas_LolE
Acinetobacter_LolE
Prim. cons.

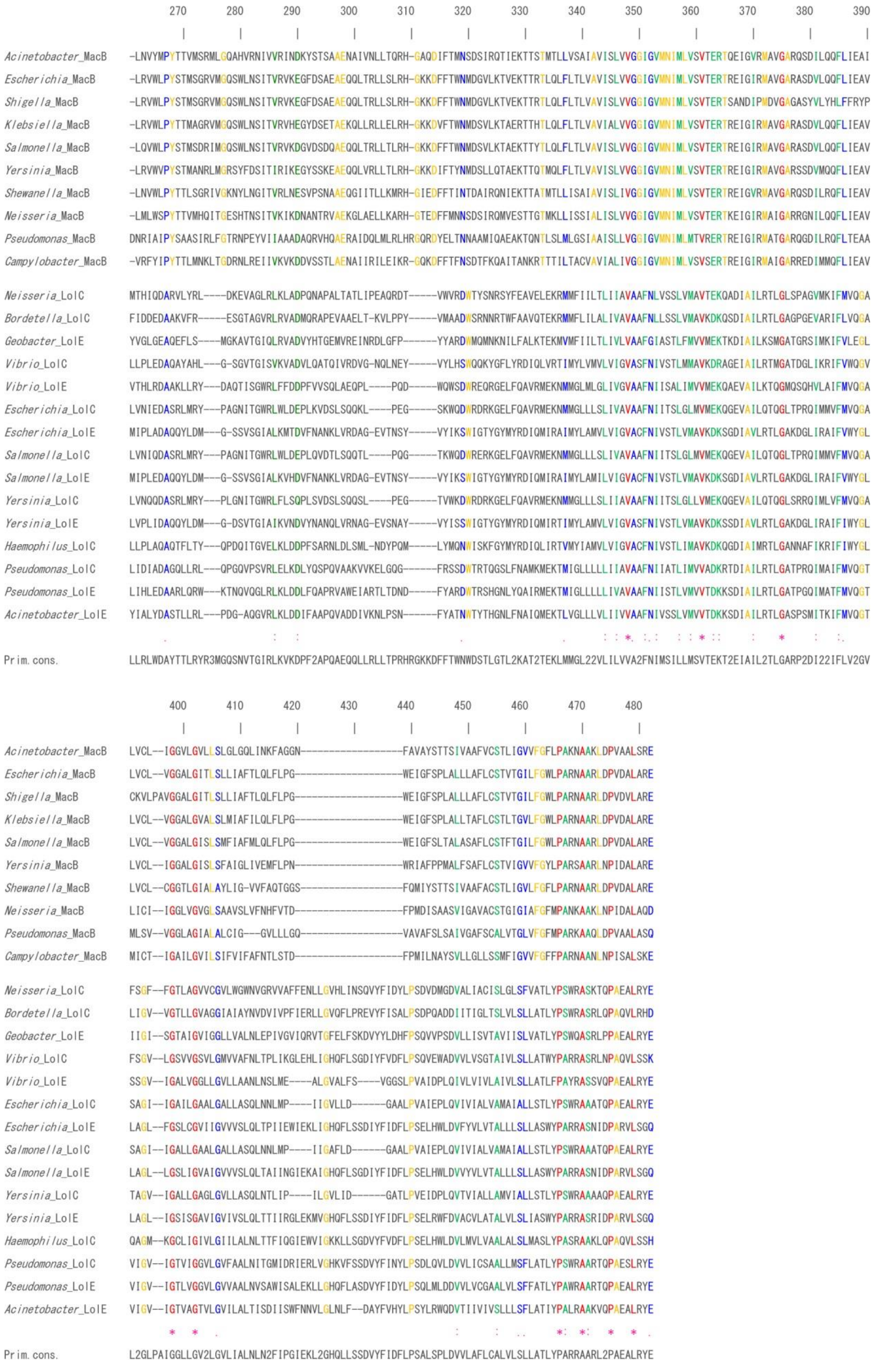
270 280 290 300 310 320 330 340 350 360 370 380 390
Acinetobacter_MacB
Escherichia_MacB
Shigella_MacB
Klebsiella_MacB
Salmonella_MacB
Yersinia_MacB
Shewanella_MacB
Neisseria_MacB
Pseudomonas_MacB
Campylobacter_MacB
Neisseria_LolC
Bordetella_LolC
Geobacter_LolE
Vibrio_LolC
Vibrio_LolE
Escherichia_LolC
Escherichia_LolE
Salmonella_LolC
Salmonella_LolE
Yersinia_LolC
Yersinia_LolE
Haemophilus_LolC
Pseudomonas_LolC
Pseudomonas_LolE
Acinetobacter_LolE
Prim. cons.
400 410 420 430 440 450 460 470 480

Supplementary Figure 12. Sequence alignment between the TMD regions of MacB and LolCD$_2$E, referred to as LolC and LolE. Sequence of NBD and connecting linker to TM1 of MacB is removed for an appropriate comparison. Letters in red, green, and blue are identical, strongly similar and weakly similar amino acids as calculated by the ClustalW multiple sequence alignment[12]. Letters in orange show identical amino acids within the MacB and LolCE families. Although these families share some strongly conserved regions at the primal sequence level, they share relatively weak overall sequence conservation. However, a strong similarity exists at the protein structure motif level.

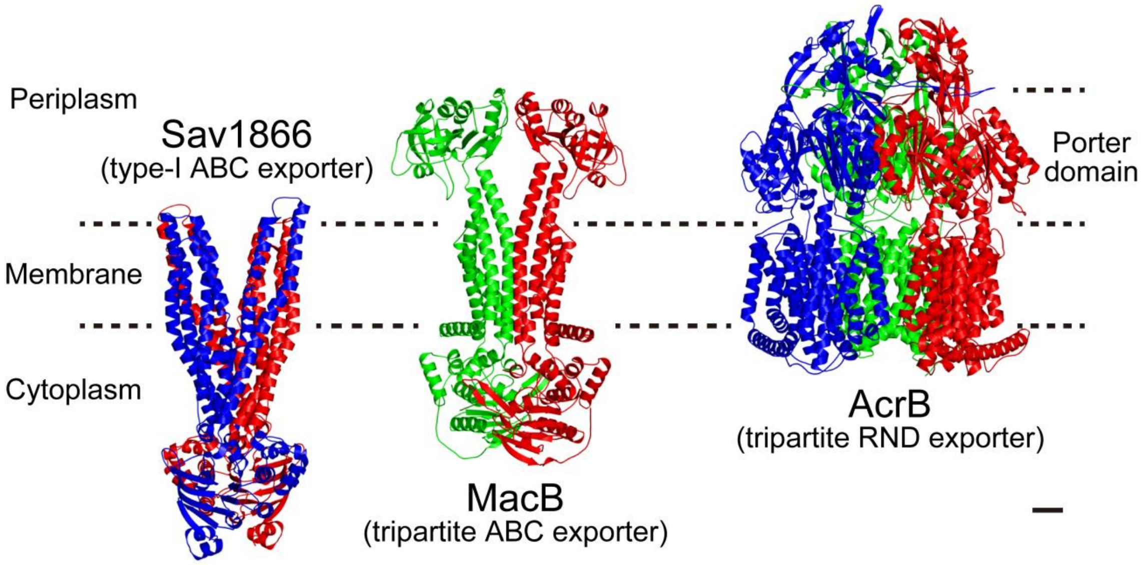

Supplementary Figure 13. Structural comparison between MacB dimer (tripartite ABC exporter), Sav1866 dimer (type-I ABC exporter, PDB accession code: 2HYD)[13] and AcrB trimer (tripartite RND exporter, PDB accession code: 4DX5)[14].

Monomers are individually coloured for each transporter, and are drawn to the same scale. The MacB PLD has a similar size as the porter domain of RND exporters, which is important for substrate recognition and translocation. Scale bar, 1nm.

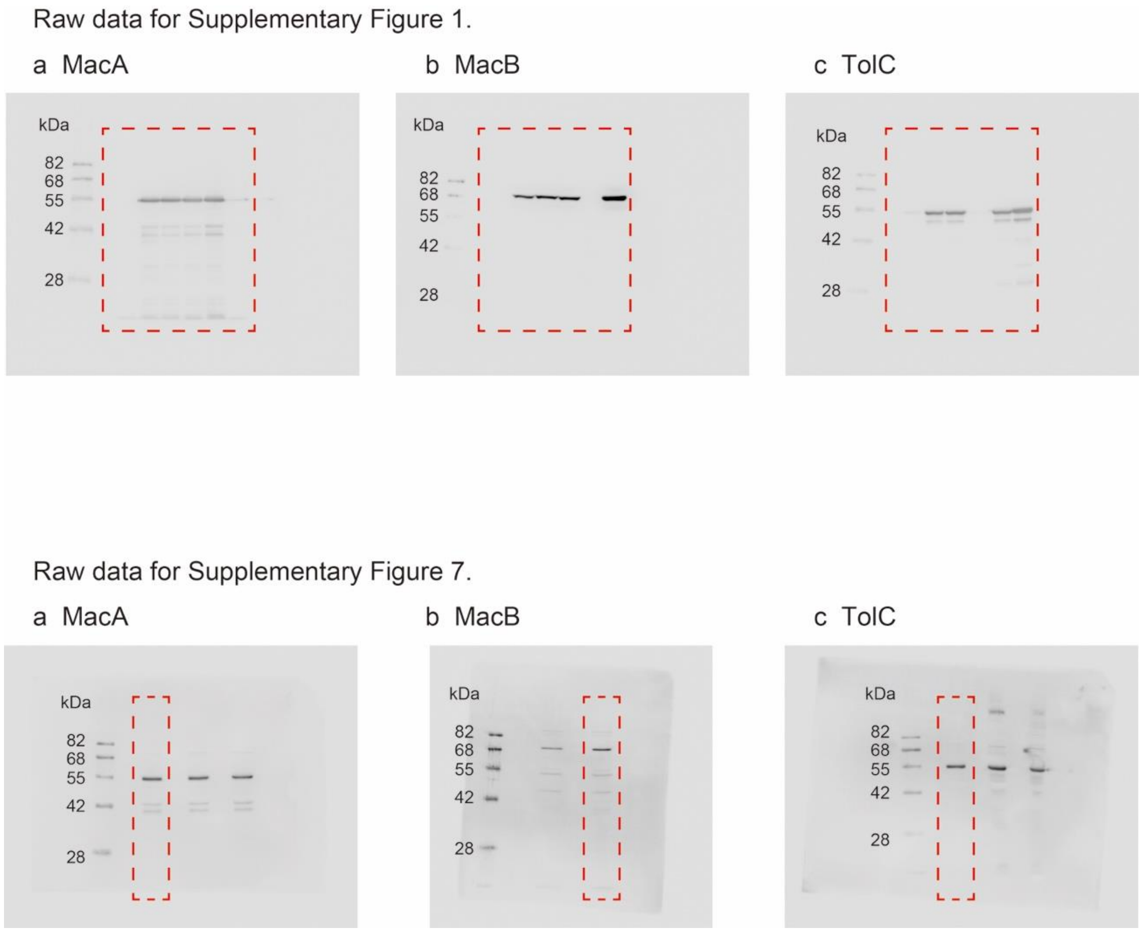

Supplementary Figure 14. Uncropped full scans of immunoblot images shown in Supplementary Figs 1 and 7. Cropped areas are marked by red boxes. Raw image data have also been deposited in the University of Cambridge data repository (https://doi.org/10.17863/CAM.12992).

Supplementary Table 1. Spatial displacement of MacB dimer NBDs when compared with ABC transporter NBDs in different nucleotide-bound states (PDB accession codes between brackets).

XYZ displacement (Å)	Dimer vs dimer[a]		Monomer-A vs monomer-A[b]	
	RMSD	average	RMSD	Average
AMP-PNP bound Sav1866 (2ONJ)	10.16	6.50	8.65	6.12
AMP-PNP bound MsbA (3B60)	10.10	6.47	8.71	6.26
ADP bound PglK (5C73)	6.28	5.17	5.46	4.44
Closed Apo MsbA (3B5X)	13.34	12.21	9.06	6.66
Open Apo MsbA (3B5W)	25.17	24.67	8.69	6.14

a, Displacement is expressed as the root-mean-square deviation (RMSD) of Cα atoms and averaged displacement of Cα atoms (Å).

b, Monomer A refers to right-hand side NBD in NBD dimers in Figure 4c.

Spatial displacement of NBDs of MacB dimer are calculated using program LSQKAB distributed with the CCP4 software package[15].

Supplementary Table 2. Structural homologues of four helix bundle of MacB (TM1: 282-321 and TM2-4: 521-664, including two CHs).

#	PDB-ID,Chain	Z	rmsd	lali	nres	%id	PDB Description
1	3g60-A	7.7	5.9	163	1182	10	MOLECULE: MULTIDRUG RESISTANCE PROTEIN 1A;
2	3g60-B	7.6	6.0	165	1182	10	MOLECULE: MULTIDRUG RESISTANCE PROTEIN 1A;
3	3g61-B	7.5	5.9	162	1182	9	MOLECULE: MULTIDRUG RESISTANCE PROTEIN 1A;
4	3g61-A	7.4	5.9	162	1182	9	MOLECULE: MULTIDRUG RESISTANCE PROTEIN 1A;
5	3wmg-A	7.4	5.4	147	589	12	MOLECULE: ATP-BINDING CASSETTE, SUB-FAMILY B, MEMBER 1;
6	4myc-C	7.3	5.9	150	597	9	MOLECULE: IRON-SULFUR CLUSTERS TRANSPORTER ATM1, MITOCHONDR
7	4myh-C	7.2	5.9	147	597	9	MOLECULE: IRON-SULFUR CLUSTERS TRANSPORTER ATM1, MITOCHONDR
8	3g5u-A	7.1	5.7	153	1182	9	MOLECULE: MULTIDRUG RESISTANCE PROTEIN 1A;
9	4m1m-A	7.1	5.7	151	1188	9	MOLECULE: MULTIDRUG RESISTANCE PROTEIN 1A;
10	4q9i-A	7.0	5.9	155	1184	7	MOLECULE: MULTIDRUG RESISTANCE PROTEIN 1A;
11	4q9h-A	6.9	5.8	149	1182	9	MOLECULE: MULTIDRUG RESISTANCE PROTEIN 1A;
12	4xwk-A	6.8	5.9	153	1182	8	MOLECULE: MULTIDRUG RESISTANCE PROTEIN 1A;
13	5c76-B	6.8	7.2	153	564	7	MOLECULE: WLAB PROTEIN;
14	5c76-A	6.8	7.2	156	564	6	MOLECULE: WLAB PROTEIN;
15	4aa3-A	6.7	6.1	152	560	9	MOLECULE: ATP-BINDING CASSETTE SUB-FAMILY B MEMBER 10,
16	4ayw-A	6.5	6.0	145	560	10	MOLECULE: ATP-BINDING CASSETTE SUB-FAMILY B MEMBER 10;
17	4q9l-A	6.4	5.7	150	1187	7	MOLECULE: MULTIDRUG RESISTANCE PROTEIN 1A;
18	4q9j-A	6.4	5.7	150	1187	7	MOLECULE: MULTIDRUG RESISTANCE PROTEIN 1A;
19	4ksd-A	6.4	6.0	153	1182	8	MOLECULE: MULTIDRUG RESISTANCE PROTEIN 1A;
20	5c76-C	6.4	7.2	153	564	7	MOLECULE: WLAB PROTEIN;
21	4q9k-A	6.3	5.9	152	1187	8	MOLECULE: MULTIDRUG RESISTANCE PROTEIN 1A;
22	4ry2-A	6.3	6.5	147	701	7	MOLECULE: ABC-TYPE BACTERIOCIN TRANSPORTER;
23	3g5u-B	6.1	5.8	160	1182	10	MOLECULE: MULTIDRUG RESISTANCE PROTEIN 1A;
24	5c78-A	6.1	6.5	150	564	7	MOLECULE: ATP-DRIVEN FLIPPASE PGLK;
25	5c78-C	6.1	6.9	153	564	7	MOLECULE: ATP-DRIVEN FLIPPASE PGLK;
26	4s0f-A	6.0	6.1	145	565	8	MOLECULE: ABC-TYPE BACTERIOCIN TRANSPORTER;
27	5c78-B	6.0	6.7	154	564	6	MOLECULE: ATP-DRIVEN FLIPPASE PGLK;
28	5c78-D	6.0	6.7	151	564	7	MOLECULE: ATP-DRIVEN FLIPPASE PGLK;
29	4mrn-B	6.0	6.0	150	598	11	MOLECULE: ABC TRANSPORTER RELATED PROTEIN;
30	4ry2-B	5.9	5.9	140	699	8	MOLECULE: ABC-TYPE BACTERIOCIN TRANSPORTER;

Top 30 hits from DALI[8,9] search, ranked by Z-score, show the structural motif conservation between MacB and mouse multidrug resistance P-glycoprotein ABCB1a (refined structure; PDB accession code: 4M1M)[16]. The following abbreviations are used. PDB-ID, chain: PDB accession code and chain identifier. Z-score: Statistical significance of the similarity between protein-of-interest and other homologous proteins. RMSD: RMS deviation of Cα atoms in the least-squares superimposition of the structurally equivalent Cα atoms. lali: number of structurally equivalent residues. nres: the total number of amino acid residues in the hit proteins. %id: percentage of identical amino acids over all structurally equivalent residues. PDB Description: the COMPND (compound) record from the PDB entry.

Supplementary Table 3. List of primers

Primers for gene amplification		
Gene	Strand	Sequence (5'-3')
macB	Forward	ggaattccatatgacaaaacaagctttgcttgaagtc
	Reverse	cgggatccttattctcgtgatagtgctgcaacag
macA-macB-tolC	Forward	catgccatgggcccaaaaataaagccaattaaactcgtaattattgtc
	Reverse	ccgctcgagttattgcggcagttcttttacaggtg
macA-macB	Forward	catgccatgggcccaaaaataaagccaattaaactcgtaattattgtc
	Reverse	ccgctcgagttattctcgtgatagtgctgcaacagg
macB-tolC	Forward	catgccatgggcacaaaacaagctttgcttgaagtcag
	Reverse	ccgctcgagttattgcggcagttcttttacaggtg

Primers for site-directed mutagenesis		
Mutation	Strand	Sequence (5'-3')
E172Q in *macB*	Forward	gaccaaccaacaggggcactcgattc
	Reverse	ggccaagatcacatcgccgccattc
Truncation of CH2 of *macB*	Forward	caaaataagaccctgttgcagcactatcacgag
	Reverse	ctgcattcttggcaggtaagaagcc
Deletion of *macB* from *macA-macB-tolC* (for *macA-tolC*)	Forward	gaaaattgcatgtctttttctatgactaaacttagcg
	Reverse	cttacattcccattgggccacggcg

Supplementary References

1 Eisenberg, D. *et al*. Analysis of membrane and surface protein sequences with the hydrophobic moment plot. *J. Mol. Biol.* **179**, 125-142 (1984).

2 Dawson, R. J. & Locher, K. P. Structure of the multidrug ABC transporter Sav1866 from Staphylococcus aureus in complex with AMP-PNP. *FEBS Lett.* **581**, 935-938 (2007).

3 Choudhury, H. G. *et al*. Structure of an antibacterial peptide ATP-binding cassette transporter in a novel outward occluded state. *Proc. Natl Acad. Sci. USA* **111**, 9145-9150 (2014).

4 Hohl, M. *et al*. Crystal structure of a heterodimeric ABC transporter in its inward-facing conformation. *Nat. Struct. Mol. Biol.* **19**, 395-402 (2012).

5 Xu, Y. *et al.* Crystal structure of the periplasmic region of MacB, a noncanonic ABC transporter. *Biochemistry* **48**, 5218-5225 (2009).

6 Xu, Y. *et al*. The Crystal Structure of the YknZ Extracellular Domain of ABC Transporter YknWXYZ from Bacillus amyloliquefaciens. *PLoS One* **11**, e0155846 (2016).

7 Abendroth, J., Mayclin, S. J., Lorimer, D. D., & Edwards, T. E. Structure of the N-terminal domain of lipoprotein-releasing system transmembrane protein LolE from *Acinetobacter baumannii*. Protein Data Bank, doi:10.2210/pdb5udf/pdb (2017).

8 Holm, L. *et al*. Searching protein structure databases with DaliLite v.3. *Bioinformatics* **24**, 2780-2781 (2008).

9 Holm, L. *et al*. Using Dali for structural comparison of proteins. *Curr Protoc Bioinformatics.* **Chapter 5,** Unit 5 5 (2006).

10 Finn, R. D. *et al*. The Pfam protein families database: towards a more sustainable future. *Nucleic Acids Res*. **44**, D279-285 (2016).

11 Hirokawa, T., Boon-Chieng, S. & Mitaku, S. SOSUI: classification and secondary structure prediction system for membrane proteins. *Bioinformatics.* **14**, 378-379 (1998).

12 Larkin, M. A. *et al.* Clustal W and Clustal X version 2.0. *Bioinformatics* **23**, 2947-2948 (2007).

13 Dawson, R. J. & Locher, K. P. Structure of a bacterial multidrug ABC transporter. *Nature* **443**, 180-185 (2006).

14 Eicher, T. *et al.* Transport of drugs by the multidrug transporter AcrB involves an access and a deep binding pocket that are separated by a switch-loop. *Proc. Natl Acad. Sci. USA* **109**, 5687-5692 (2012).

15 Winn, M. D. *et al.* Overview of the CCP4 suite and current developments. *Acta Crystallogr. D* **67**, 235-242 (2011).

16 Li, J., Jaimes, K. F. & Aller, S. G. Refined structures of mouse P-glycoprotein. *Protein Sci.* **23**, 34-46 (2014).
