# Supplementary material for: Crystal structure of tripartite-type ABC transporter MacB from Acinetobacter baumannii
Source: Nat Commun. 2017 Nov 6;8:1336. doi: 10.1038/s41467-017-01399-2 (PMC5673888; doi:10.1038/s41467-017-01399-2)
Supplement: Supplementary file 3 — Description of Additional Supplementary Files [file 41467_2017_1399_MOESM3_ESM.pdf]

Description of Additional Supplementary Files

File Name: Supplementary Movie 1
Description: **Superimposition of NBDs of MacB and typical type-I exporter Sav1866**. With the NBDs superimposed, the two CHs for each transporter are also superimposed. However, the order of the CHs and connecting TMs are different.

File Name: Supplementary Movie 2
Description: **Structural comparison between outward-facing conformations of AMP-PNP-bound Sav1866 (left: PDB accession code: 2ONJ) and MacB (right) with hydrophobicity scale from white to red, as described for Supplementary Figure 3.**
